# Nuclear magnetic resonance evaluation of inflammatory activity from chronic viral Hepatitis B

**DOI:** 10.12669/pjms.35.6.1364

**Published:** 2019

**Authors:** Jinghui Dong, Yuan Liu, Huiyi Ye, Weimin An, Hongwei Ren

**Affiliations:** 1 Jinghui Dong, Department of Radiology, Medical School of Chinese PLA, Beijing 100853, P. R. China. Department of Radiology, The Fifth Medical Center of Chinese PLA General Hospital, Beijing 100039, P. R. China; 2 Yuan Liu, Department of Radiology, The Fifth Medical Center of Chinese PLA General Hospital, Beijing 100039, P. R. China; 3 Huiyi Ye, Department of Radiology, The First Medical Center of Chinese PLA General Hospital, Beijing 100853, P. R. China; 4 Weimin An, Department of Radiology, The Fifth Medical Center of Chinese PLA General Hospital, Beijing 100039, P. R. China; 5 Hongwei Ren, Department of Radiology, The Fifth Medical Center of Chinese PLA General Hospital, Beijing 100039, P. R. China

**Keywords:** Hepatitis B, Inflammatory activity, Magnetic resonance imaging, Diffusion-weighted imaging

## Abstract

**Objectives:**

To discuss the value of applying magnetic resonance diffusion-weighted imaging (DWI) to evaluate inflammatory activity from chronic viral hepatitis B.

**Methods:**

One hundred forty-two patients with chronic viral hepatitis B who received treatment at The Fifth Medical Center of Chinese PLA General Hospital from January 2014 to December 2015 and 20 healthy persons in the control group who were scheduled to undergo nuclear magnetic resonance scanning and DWI examinations (b value = 0, 800 s/mm^2^), and the apparent diffusion coefficients (ADCs) were measured and compared with the biopsy results of hepatic tissue.

**Results:**

The ADC value of the group with hepatitis B was lower than that of the healthy group (P<0.05), and the ADC value of the group with mild inflammation (G1) significantly differed from that of the group with moderate inflammation (G2) and that of the group with severe inflammation (G3-G4) (P<0.05).

**Conclusions:**

Magnetic resonance diffusion-weighted imaging technology has high clinical value for evaluating the inflammatory activity from chronic hepatitis B, and the measured ADC value corresponds to the pathological grade well, so this method is worth clinical promotion and application.

## INTRODUCTION

In China, the number of carriers of hepatitis B accounts for ten percent of the total population. Chronic viral hepatitis is persistent and recurrent and ultimately develops into hepatic fibrosis, hepatic cirrhosis and even hepatic carcinoma, all of which gravely threaten patient health.^1^ Generally, lesions from chronic hepatitis B are associated with inflammation and fibrosis. The inflammation grade of hepatic tissue reflects the current status of liver injury and hepatitis activation. The hepatic fibrosis stage indicates cumulative liver injury and is related to the course of disease. If treatment is offered during this period, then the lesion can be reverted to healthy tissue.^2^

The current gold standard for evaluating the inflammation indexes of chronic hepatitis B is liver biopsy. However, liver biopsy is invasive and has various disadvantages, such as limited sampling, low patient compliance, and poor examination repeatability. Thus, a simple and noninvasive method with high repeatability is sorely needed to evaluate the inflammatory activity of chronic hepatitis B. Magnetic resonance diffusion-weighted imaging (DWI) technology has a clear advantage in diagnosing hepatic diseases. This study aimed to preliminarily discuss and compare magnetic resonance diffusion-weighted imaging and pathological grading in assessing inflammatory activity from chronic hepatitis B; DWI is hopefully of great clinical significance in guiding clinical diagnosis and treatment, therapeutic evaluations and follow-up visits for chronic hepatitis.

## METHODS

One hundred forty-two patients with chronic viral hepatitis B admitted to The Fifth Medical Center of Chinese PLA General Hospital from January 2014 to December 2015 were observed, including 89 male patients and 53 female patients. The patients were aged 18 to 67 (42.5±5.8 on average).

### Ethical Approval

The study was approved by the Institutional Ethics Committee of The Fifth Medical Center of Chinese PLA General Hospital, and written informed consent was obtained from all participants. (Approval date December 8, 2013)

All the selected patients complied with the diagnostic criteria as specified in the 2005 Guideline on Prophylaxis and Treatment of Chronic Hepatitis B,^3^ were confirmed not to have hepatitis B with hepatitis C, hepatitis D, cytomegalovirus, human papillomavirus virus, HIV, alcoholic liver disease, drug-induced hepatitis, autoimmune liver disease, or cholestatic liver disease, and were not complicated with cirrhosis or liver tumors. The patients immediately underwent liver biopsy within two days after MRI, and two associate chief physicians from the pathology department jointly read the slices, consulted with each other and determined the histopathologic grade. The severity of hepatitis was graded as G0-4 and S0-4 in accordance with the standards revised at the 2000 Xi’an National Virus Hepatitis and Liver Disease Conference.^4^ Since the number of G4 cases among this group was small, G3 and G4 were combined as severe inflammation. On this basis, liver inflammation activity was classified as mild (G1), moderate (G2) and severe (G3, G4). Moreover, 20 healthy persons were selected as the control group, and these patients had no pre-existing liver disease, had normal hepatic function indexes and were aged 30 to 56 (46.1±3.2 on average). All the selected patients were approved by the hospital ethics committee, and the patients were asked to sign informed consent Forms.

### Examination Method

The selected patients received conventional MR scanning and DWI scanning. For the MRI examination, a GE HD x 3.0T high-intensity magnetic resonance imaging system and an 8-channel phased-array body surface coil were adopted, and the scanned area was from the diaphragmatic dome to the inferior margin of the liver. DWI scanning was followed by axial T1WI and T2WI scanning. The scanning parameters were as follows: TR 1900 ms, TE 64 ms, b=800 mm^2^/s, slice thickness 8 mm, interlayer spacing 1 mm, view 38 cmx38 cm, matrix 128x128, number of excitations 2, number of scanning layers 20, and scan time 29 s; the diffusion direction was all directions, breathless collection was conducted on the end-expiratory row, and two breath holding cycles were conducted to complete scanning.

### ADC Value Measurement

The ADC values of three regions of interest of the same size in different locations on each of the three consecutive layers of the right rear lobe of the liver were measured with the Function tool of the software in the AW4.5 workstation equipped with a GE HDxt3.0T MR machine. The area of the region of interest was approximately 200-300 mm^2^, and blood vessels, bile ducts, and chemical shift artifacts were avoided; the average of the nine values was taken as the observed ADC value of the liver.

### Grading Standard

4 The severity of hepatitis was graded and staged via the Knodell HAI score (Table-I).

**Table I:** Criteria for staging and grading chronic hepatitis (2000, Xi’an).

Inflammatory activity		Degree of fibrosis		
Grade	Portal area and periphery	Intralobular	Stage	Fibrosis
G0	No inflammation	No inflammation	S0	None
G1	Portal area inflammation	Degeneration and a little focal necrosis	S1	Portal area expansion, fibrosis
G2	Mild piecemeal necrosis	Degeneration, spotted and focal necrosis or acidophilic body	S2	Fibrous septum is forming, and lobular structure is retained
G3	Moderate piecemeal necrosis	Degeneration, severe necrosis or visible bridging necrosis	S3	Fiber insulation with lobular disorder, without liver cirrhosis
G4	Severe piecemeal necrosis	Wide area of bridging necrosis to involve multiple lobules, lobular structure disorder	S4	Forepart hepatocirrhosis

SPSS 21.0 software was applied for data processing and analysis, and the mean±standard deviation (χ±s) was used to denote measurement data; variance analysis was adopted for intergroup comparisons, the LSD-q test was adopted for intragroup pairwise comparisons, and P<0.05 indicated statistical significance. The receive operator characteristic (ROC) curve was used to evaluate the efficiency of diagnosing liver G2 or higher inflammation via the ADC value.

## RESULTS

**Fig. 1:**
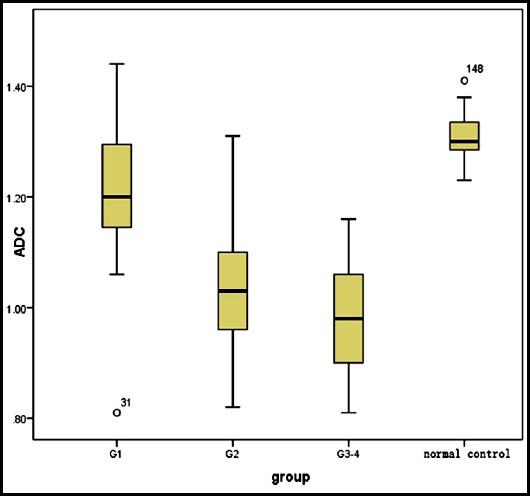
ADC values of groups with different grades of inflammation from Chronic Hepatitis B and the Normal Control Group (x10-3 mm2/s).

The box plots of the normal control group and different grades of inflammatory activity from chronic hepatitis B when b=800 s/mm^2^ are shown in Fig. 1. The ADC values of both the moderate (G2) and severe (G3, G4) inflammation groups were significantly lower than those of the mild (G1) inflammation and normal control groups (G0) (P<0.05) (Table-II).

**Table II:** ADC values of the normal control group and groups with different grades of inflammation from Chronic Hepatitis B (x10-3 mm2/s).

	Control group(G0)	Mild (G1)	Moderate (G2)	Severe (G3, G4)
N	20	43	61	38
ADC value	1.31±0.16	1.22±0.12	1.05±0.12*	0.98±0.10*

## DISCUSSION

The overall objectives of treating patients with hepatitis B according to the Guideline on Prophylaxis and Treatment of Chronic Hepatitis B are to inhibit HBV for as long as possible, alleviate hepatocyte inflammatory necrosis and hepatic fibrosis, to delay and reduce liver decompensation, liver cirrhosis, hepatocellular carcinoma and complications thereof, to improve patient quality of life and to prolong survival time. Both domestically and internationally, it is universally considered that the key to delaying the progression of chronic hepatitis B is antiviral therapy. Sometimes, clinical and laboratory examinations are not reliable for guiding clinical treatment. Thus, pathological results are needed to guide clinic treatment, and it is noted that an ALT≥2ULN or inflammation graded as G2 or higher and/or as S2 or higher is an indicator to initiate antiviral therapy.^5^ Patients with an ALT<2ULN usually have no obvious symptoms, which are often ignored by both doctors and the patients themselves. For such patients, antiviral therapy is recommended once liver inflammation reaches grade G2. According to previous studies,^6^,^7^ the proportion of patients with chronic hepatitis B with an ALT<2ULN suffering from obvious inflammation and hepatic fibrosis is large. Presently, liver biopsy is the gold standard for evaluating the inflammatory activity of chronic hepatitis B. However, liver biopsy is invasive, and the sampling coverage is limited. Thus, the degree of inflammation in the whole liver cannot be evaluated. In addition, patients with severe inflammation are not appropriate candidates to undergo liver biopsy. Thus, liver biopsy is not a good method for dynamically monitoring the progression of inflammation severity and evaluating curative effects. Therefore, a noninvasive method with high repeatability is needed to evaluate the severity of inflammation in the liver.

Early diagnosis of diffuse hepatopathy is of great significance for guiding clinical treatment and evaluating curative effects but is rarely detected with imaging, which is mainly applied for observing anatomical structure and morphological changes.^8^ In recent years, with the development of magnetic resonance technology, DWI has emerged as a noninvasive functional imaging technology to analyze the interior structures and tissue elements of lesions based on the diffusion motion of water molecules. The basic lesions of chronic hepatitis B feature inflammation and fibrosis. Pathologically, inflammation grade and fibrosis severity represent the current injury status of the liver and cumulative injury of inflammation, respectively, which are related to the duration of inflammatory injury. Inflammation of hepatic tissue will develop into hepatic fibrosis to a certain extent if immediate treatment is not conducted, and early hepatic fibrosis can recover with treatment. Therefore, the early diagnosis and treatment of this disease are of great importance. It was reported^9^ that inflammatory necrosis of hepatic tissue may result in an increase in portal vascular resistance, slow down the blood velocity of the portal vascular, and further cause hepatic fibrosis to develop into hepatic cirrhosis. Hepatic cirrhosis is almost irreversible. Therefore, the progression of chronic hepatitis B can be delayed by controlling changes in liver inflammation, which is of great significance for guiding subsequent clinical treatment and affecting the prognosis at follow-up.

The ADC value varies with the b value. Thus, it is important to apply a proper b value. The liver is a solid organ with rich blood circulation, so hemoperfusion will affect the accuracy of the ADC measurements. Studies have shown that a small b value leads to a high image SNR and a clear image; however, hemoperfusion has a great impact on the ADC value because the diffusion motion of water molecules cannot be reflected well, so the measured ADC value is overestimated. The impact of hemoperfusion is negatively correlated with the b value; a large b value will lead to a relatively accurate ADC value,^10^ but this relationship is not always the case. In the case of an excessively large b value, the image SNR is drastically reduced, the artifact is large, and the measured ADC value is not accurate.^11^ To sum up, when b=800 s/mm^2^, hemoperfusion has a slight impact, and the diffusion motion of water molecules can be truly reflected, which means that clear images are obtained, and the measured ADC value is stable.

The ADC value of the liver is determined by liver inflammation and fibrosis. Most previous reports did not include layering research on these two aspects, and the impact of hepatic fibrosis on the ADC value was not excluded, so the measured ADC value was relatively inaccurate. The studies by Ying et al.^6^ and Li-tao S^12^ show that there exists a significant difference in ADC value between S0 and S3, S0 and S4, S1 and S3, and S1 and S4, while the difference among S0, S1 and S2 is not statistically significant. The fibrosis stages of the observed patients in this study ranged from S0 to S2, so S3 and S4 were excluded. In this way, the measured deviation caused by the impact of fibrosis on the ADC value of hepatic inflammatory activity can be avoided as much as possible. Moreover, the ADC value of the liver is affected by fatty infiltration and iron deposition. Since iron is a paramagnetic substance, iron deposition will shorten the T2 time of the liver to impact the quality of the DWI image and lead to inaccurate ADC values. Fatty infiltration causes the number of free protons in the liver to increase and affects the accuracy of the ADC value. For the purpose of this study, patients with fatty livers and iron overload were excluded so that the patients with chronic hepatitis B would pass the DWI evaluation of inflammatory severity and that this method provides quantitative reference indexes for clinical treatment and prognosis evaluations.

**Fig. 2:**
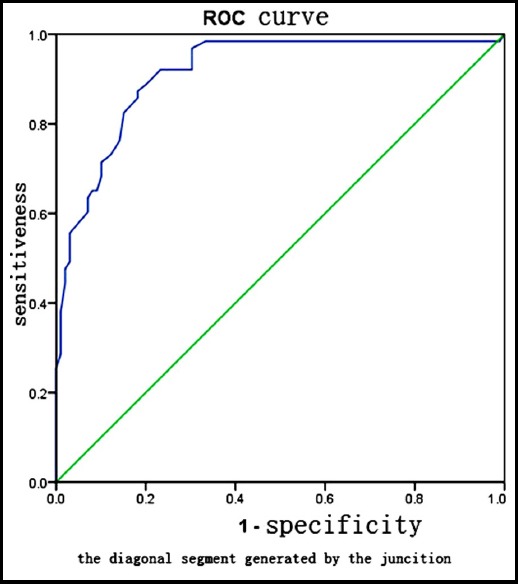
ROC Curve of G2 or Higher Inflammation Activity in Patients with Chronic Hepatitis B diagnosed via the ADC Value.

This study suggests that when b=800 s/mm^2^, the differences between mild (G1) and moderate (G2) inflammatory activity and between mild (G1) and severe (G3, G4) inflammatory activity are statistically significant. According to the therapeutic standards specified in the Guideline on Prophylaxis and Treatment of Chronic Hepatitis B, patients with G2 or higher inflammation should receive antiviral therapy. Thus, patients G2 or higher inflammation were taken as the inflammation group in this study, and those patients with G0-1 inflammation were taken as the non-inflammation group. According to the characteristics of the ROC curve (Fig. 2), the area below the ROC curve reaches a maximum when ADC=1.13, and a high accuracy is achieved when the AUC=0.914; the sensitivity and specificity of the inflammation group and non-inflammation group are 87.3% and 81.8%, respectively. When the ADC is >1.13, patients are diagnosed with G0-1 inflammation. In such cases, no antiviral therapy is needed if the ALT is normal. When the ADC is ≤1.13, patients are diagnosed with G2 or higher inflammation, and the patients should actively take antiviral therapy. In other words, an ADC=1.13 is regarded as the best critical value for initiating antiviral therapy.

### Limitations of the study

First, there are differences between the liver biopsy method and the method for measuring ADC values. To address this problem, an ROI of the right rear lobe of the liver was selected to measure the ADC value to be as consistent as possible with the biopsy method. Second, a single b value was applied to research the degree of inflammatory necrosis of the liver to avoid balancing the impacts of hemoperfusion and diffusion on the ADC value. Thus, multiple b values should be applied to ensure the accuracy of the measurement, which will be further discussed and studied in the future. Although DWI can be regarded as a quantitative analysis method, this study suggests that the differences in ADC value between the hepatitis group and normal control group and between the inflammation group and non-inflammation group are significant and can help make clinical antiviral therapeutic schemes, provided that the other clinical laboratory indexes are referred to.

In summary, magnetic resonance diffusion-weighted imaging can reflect early diffuse lesions of the liver well, ADC measurements can help with the diagnosis, and dynamic observations of the ADC value can reveal the progression of inflammatory activity in the liver and the therapeutic effect; DWI has high application value in evaluating the inflammatory activity of patients with chronic hepatitis B and is worth clinical promotion and application.

### Author Contributions:

**JD &** **YL:** Collected and analyzed clinical data and prepared this manuscript, are responsible for integrity of research.

**HY:** Designed this study.

**WA &** **HR:** Significantly revised this manuscript.
